# A two-phase substrate model for enzymatic hydrolysis of lignocellulose: application to batch and continuous reactors

**DOI:** 10.1186/s13068-019-1633-2

**Published:** 2019-12-27

**Authors:** James J. Lischeske, Jonathan J. Stickel

**Affiliations:** 1 National Bioenergy Center, National Renewable Energy Laboratory, 15013 Denver West Pkwy, Golden, CO USA; 2 Biosciences Center, National Renewable Energy Laboratory, 15013 Denver West Pkwy, Golden, CO USA

**Keywords:** Enzymatic-hydrolysis modeling, Continuous enzymatic hydrolysis

## Abstract

**Background:**

Enzymatic hydrolysis continues to have a significant projected production cost for the biological conversion of biomass to fuels and chemicals, motivating research into improved enzyme and reactor technologies in order to reduce enzyme usage and equipment costs. However, technology development is stymied by a lack of accurate and computationally accessible enzymatic-hydrolysis reaction models. Enzymatic deconstruction of cellulosic materials is an exceedingly complex physico-chemical process. Models which elucidate specific mechanisms of deconstruction are often too computationally intensive to be accessible in process or multi-physics simulations, and empirical models are often too inflexible to be effectively applied outside of their batch contexts. In this paper, we employ a phenomenological modeling approach to represent rate slowdown due to substrate structure (implemented as two substrate phases) and feedback inhibition, and apply the model to a continuous reactor system.

**Results:**

A phenomenological model was developed in order to predict glucose and solids concentrations in batch and continuous enzymatic-hydrolysis reactors from which liquor is continuously removed by ultrafiltration. A series of batch experiments were performed, varying initial conditions (solids, enzyme, and sugar concentrations), and best-fit model parameters were determined using constrained nonlinear least-squares methods. The model achieved a good fit for overall sugar yield and insoluble solids concentration, as well as for the reduced rate of sugar production over time. Additionally, without refitting model coefficients, good quantitative agreement was observed between results from continuous enzymatic-hydrolysis experiments and model predictions. Finally, the sensitivity of the model to its parameters is explored and discussed.

**Conclusions:**

Although the phenomena represented by the model correspond to behaviors that emerge from clusters of mechanisms, and hence a set of model coefficients are unique to the substrate and the enzyme system, the model is efficient to solve and may be applied to novel reactor schema and implemented in computational fluid dynamics (CFD) simulations. Hence, this modeling approach finds the right balance between model complexity and computational efficiency. These capabilities have broad application to reactor design, scale-up, and process optimization.

## Background

As one of several process steps in the biochemical conversion of biomass to fuels and chemicals, enzymatic hydrolysis (EH) continues to contribute significantly to the total conversion cost. Traditionally, the unit operation (process step) of EH has been performed and analyzed as a batch operation [6], but, more recently, there has been interest in alternative conversion strategies in order to reduce enzyme usage and capital costs through process intensification [8, 22, 31, 34]. However, systematic development and economical evaluation of these novel processes are stymied by a lack of accessible reaction-kinetics models.

Hydrolysis of lignocellulose via a vendor-provided cocktail of enzymes is a very complex and heterogeneous reaction. Capturing all the physico-chemical mechanisms in a single comprehensive model for a single well-mixed batch reactor is a very difficult conceptual task, and simulation of these models requires high computational costs [11, 18]. When combined with advanced reactor schemes, e.g., multiple CSTRs, or with transport models, e.g., coupled computational fluid dynamics (CFD) simulations, the use of a comprehensive model requires high-performance computing resources or may be effectively intractable with current computers.

While mechanistic (i.e., physics- and chemistry-based) models are preferred by scientists and engineers, much can be gained from models that are phenomenological (representing observed macroscopic phenomena) or empirical (reproducing measured data). These models may be constructed with mathematical expressions that have more relaxed requirements on their form and hence are often much more computationally efficient. As long as these models faithfully predict the phenomena of interest, they may be used for engineering calculations in the design of products and processes.

There have been a plethora of phenomenological/empirical models proposed in the literature for the enzymatic hydrolysis of (ligno-)cellulose. Bansal et al. [2] cite more than 70 in their review. Modeling efforts have hardly diminished in recent years, although the focus has shifted more towards mechanistic models. Many of the models are designed to reproduce the rate slowdown, employing a wide variety of approaches, and it is clear that the reduction in hydrolysis rate is due to multiple factors. The most easily modeled is enzyme inhibition due to unproductive complexes with product sugars and other species like lignin. Yet, even accounting for inhibition, a marked rate slowdown is observed during the enzymatic hydrolysis of lignocellulose [29].

Modeling approaches to account for this inherent rate reduction may be first classified as either enzyme- or substrate-based. Enzyme-based rate slowdown is often accomplished by including a rate term for enzyme deactivation [14, 25]. Thermal destabilization and mixing shear have been advanced as hypotheses justifying this approach [7], although these mechanisms are insufficient to describe slowdown of hydrolysis performed at low temperature and under limited mixing. A more plausible case has been made that enzymes get “stuck” and are no longer productive [9, 10]. However, there is evidence that this is a temporary phenomenon, and the enzymes are still active when exposed to fresh substrate [34].

Another, more compelling, possibility is that the rate reduction is due to substrate properties, although the precise mechanisms are not yet clearly understood. Bansal et al. [3] performed a systematic study and found that approximately 90% of the cause of rate retardation was due to substrate depletion, accessibility, and “hydrolysability” (propensity of cellulose to be hydrolyzed by enzymes), while the intrinsic reactivity remained effectively unchanged. Olsen et al. [21] found a correlation between hydrolysis rate and surface area (and roughness) of cellulose particles, which decrease with the extent of conversion.

Several modeling approaches have been used to implement hydrolytic rate reduction due to substrate properties. Some have implemented a substrate “reactivity” parameter that decreased with fractional conversion [12]. Another interesting approach is the use of so-called fractal kinetics to describe the progression of EH on spatially confined (ligno-)cellulosic substrates [33, 36]. However, both of these approaches (substrate reactivity and fractal kinetics) require knowledge of the extent of conversion of the substrate. While this is trivial when evaluating simple well-mixed batch reactions, it is harder to implement in advanced reactors (e.g., multiple continuous reactor systems) and in coupled CFD simulations. Liang et al. [14] resolved this difficulty for a countercurrent unit operation by applying a Continuum Particle Distribution Modeling (CPDM) approach to a countercurrent saccharification process. CPDM theory was developed by Loescher [16], who derived equations for several different process configurations. Similarly, Tervasmaki et al. [32] modeled fed-batch reaction by discretizing the substrate according to its time in the reactor while forcing several kinetic properties to change with increasing conversion. However, these approaches do not seem to be portable or flexible, requiring either re-derivation of the underlying systems of equations for new applications or implementation of a computationally expensive population balance model. Therefore, we prefer rate-based kinetics models that depend only on state variables without requiring knowledge of past history.

Many mechanistic models emphasizing the impact of substrate properties exist, but are often too detailed to efficiently calculate in a dynamic system. For example, Levine et al. [13] modeled substrate as populations of mono- and poly-disperse spheres which reduce enzyme-available surface area over time, Luterbacher et al. [17] modeled diffusion of a generalized enzyme into a porous cylinder where it reacted with substrate to form products, Zhang et al. [37] developed a structural model of interweaving cellulose and xylose and modeled several enzyme modalities, and Nag et al. [18] considered the depolymerization of cellulose partitioned into crystalline and amorphous populations. More recently, Ahamed et al. [1] modeled cellulose particles as a heterogenous cylinder wherein both enzyme transport and cellulose degree of polymerization vary across the domain, and applied the reaction scheme to model a fed-batch reaction. While all of these models elucidate important features of enzymatic deconstruction, each of these models requires features to represent the kinetics that are computationally difficult to resolve (diffusive transport in Luterbacher et al. [17], population balance in Levine et al. [13], Nag et al. [18], and Ahamed et al. [1]), and thus are poorly suited for direct application in process design or CFD models.

Here we describe a relatively simple model for the enzymatic hydrolysis of lignocellulose. Several established reaction mechanisms are ignored, including synergistic action of component enzymes, interdependent and evolving substrate microstructure and accessibility, and the size-polydispersity of the cellulose substrate. We do consider the important macroscopic phenomenon of reaction-rate slowdown. As discussed above, this phenomenon is likely due to the complex evolution of substrate structure and morphology. Nonetheless, we assert that the rate slowdown may be sufficiently represented by using a two-phase substrate formalism, i.e., a cellulose substrate that is composed of two populations: a *facile* population that is easily digested and a *recalcitrant* population that is digested more slowly. Conceptually, we consider that the difference between these populations is due to their different accessibility to enzymes rather than a difference in kinetic rate. Adsorption and rate expressions are used for each glucan population that do not require knowledge of their history, making the model easy to implement in larger simulation systems. Interactions with lignin and xylan are also considered, including xylan hydrolysis and unproductive adsorption of enzymes on soluble lignin. A cellulose-only application of this model has previously been implemented in the context of CFD simulation of a stirred reactor, quantifying the impacts of an under-mixed hydrolysis environment [27].

We illustrate the application of this model with a continuously stirred tank reactor (CSTR) system in which liquor is continuously removed via cross-flow ultrafiltration in a pump-around loop. This advanced reactor concept has potential, compared to batch reactors, to improve rates through reduced product inhibition while retaining enzyme within the reactor system, and to provide a solids-free sugar source for downstream upgrading. This system will be described in “Materials and methods” section and has been studied previously [31], although with a less-rigorous modeling approach. Batch experiments are used as the basis for parameter determination, the model is applied to the CSTR system using the mass-flow rates calculated by the control system, and sugar concentrations and insoluble solids fraction from continuous EH experiments are compared to model predictions. Presentation of these results is followed by a sensitivity study of the model parameters.

## Results and discussion

A full discussion of the model, phenomenological features represented, and its parameters may be found in “Materials and methods: Enzymatic-hydrolysis model” section. Briefly, cellulose (glucan) is divided into recalcitrant and facile categories (where $y_{\text{F}0}$ indicates the initial mass fraction of the facile population) to reflect the biphasic character of cellulose digestion. Recalcitrant cellulose is modeled as having structure, such that enzyme-accessible recalcitrant cellulose is less than the total amount of recalcitrant cellulose. Enzyme is considered to be adsorbed in equilibrium with the various substrates and inhibitors: specifically, enzyme is partitioned between the cellulose fractions, xylan, lignin, and soluble sugars, as well as some enzyme remaining free in solution. Using equilibrium constants for adsorption to each species ($K_{\text{di}}$), $\kappa _{\text{ij}}$ parameters are derived to indicate the relative strength of each adsorption effect at equilibrium. The hydrolysis-rate parameters ($k_i$) control the rates of conversion of the xylan and glucan populations, and the solubilization of lignin.

### Fit to batch data

**Fig. 1:**
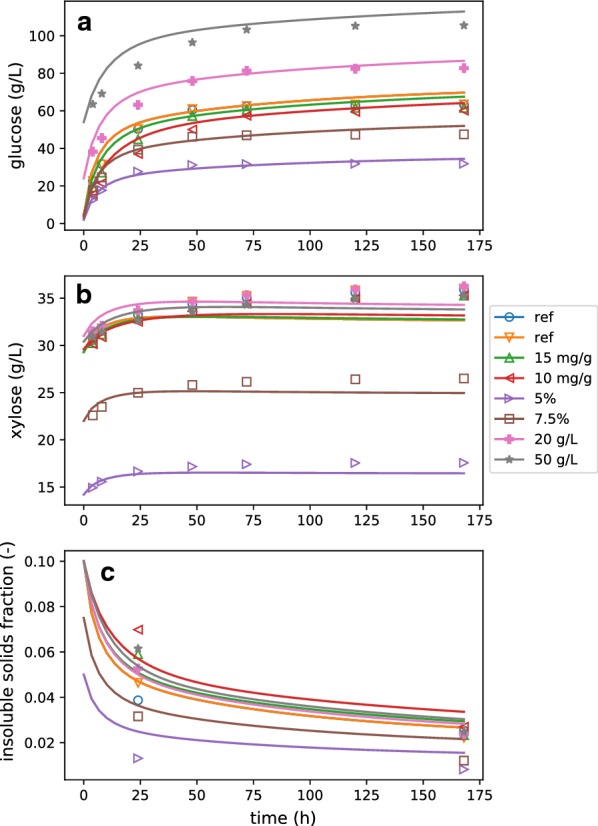
Best-fit model compared to batch experiments. Batch experiments were conducted where enzyme loading, initial insoluble solids, and background glucose were varied, and the kinetics model was fit to these data. Glucose (**a**), xylose (**b**), and insoluble solids (**c**) concentrations are shown along with the model fits for each experiment. In the legend, “ref” refers to the reference condition, 10 and $15 \,\mathrm {mg/g}$ refer to experiments where enzyme loading was reduced from $20 \,\mathrm {mg/g}$ cellulose, 5 and $7.45\%$ refer to experiments where $f_{\text{is}}$ was reduced from $10\%$, and 20 and $50 \,\mathrm {g/L}$ refer to experiments where glucose was added exogenously to the initial condition

A series of batch experiments were performed (details in Materials and methods: Batch enzymatic hydrolysis), and best-fit model parameters were determined by constrained nonlinear least-squares fitting methods (SciPy’s implementation of Nelder–Mead [19, 20]). The resulting parameters are given in Table 1, and the model fits against glucose, xylose, and insoluble solids fraction ($f_{\text{is}}$) data are shown in Fig. 1. The model predictions fit the glucose data well, with most data falling within a few percent of the model prediction. This shows that the reaction model is able to account for the effects of differing solids concentration, enzyme loading, and glucose concentration on the outcomes of enzymatic hydrolysis, at least for the range of parameters tested. We observe that the model systematically over-estimates sugar concentration for the case with $50 \,\mathrm {g/L}$ added glucose (Fig. 1a), indicating that the best-fit model may be slightly underestimating the impact of soluble sugar inhibition.

**Table 1:** Summary of the fitted model parameters

Parameter	Value	Units
$k_\text{R}=k_\text{F}$	14,713	$\text{h}^{-1}$
$k_\text{X}$	10,000	$\text{h}^{-1}$
$k_\text{L}$	729.5	$\mathrm {m^3\ liquid/kmol}$
$K_{\text{dR}}$	0.05	$\mathrm {kmol/m^3\ liquid}$
$\kappa _{\text{RF}}$	9.34	(–)
$\kappa _{\text{RL}}$	50	(–)
$\kappa _{\text{RX}}$	11.3	(–)
$\kappa _{\text{Rs}}$	50	(–)
$y_{\text{F}0}$	0.60	(–)

All parameters result from a constrained nonlinear constrained least-squares fit against the batch data

The model estimates for xylose concentration capture the initial curvature of the xylan conversion, but each experiment ended at a higher xylose concentration than the asymptotic value in the simulation (Fig. 1b). It should be noted that the pretreatment conditions were targeted towards optimal conversion of xylan to xylose monomers in order to maximize substrate reactivity during EH. This resulted in a low fraction of xylan in the solids ($w_\text{x} = 0.06$) and a liquor with significant background xylose. Thus, initial background sugar levels dominate the soluble sugars measured throughout conversion, rather than converted sugars. Based on our experience performing similar experiments, we think that the total amount of xylan available for conversion may have been underestimated by the analytical methods, hence the systematic under-estimation of xylose liberation.

The estimated end-point $f_{\text{is}}$ values agree well with the experimental data (Fig. 1c), supporting the model assumption that lignin is solubilized over the course of reaction [23]. Model simulations without lignin solubilization ($k_\text{L}=0$) resulted in much higher estimation of $f_{\text{is}}$ (results not shown). The $f_{\text{is}}$ measurements at 24 h show much more spread than the model fit. We do not know why this is, but it may suggest a mechanism for lignin solubilization that is not exactly proportional to sugar hydrolysis, as assumed here.

**Fig. 2:**
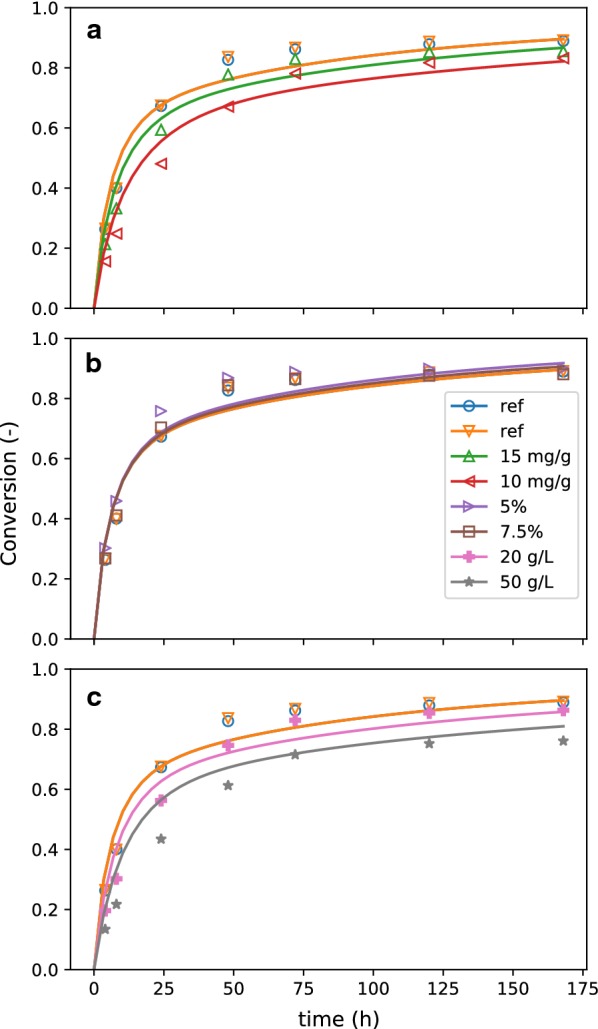
Best-fit conversion compared to batch experiments. In the legend, “ref” refers to the reference condition (shown in all three plots), 10 and $15 \,\mathrm {mg/g}$ refer to experiments where enzyme loading was reduced from $20 \,\mathrm {mg/g}$ cellulose (**a**), 5 and $7.45\%$ refer to experiments where $f_{\text{is}}$ was reduced from $10\%$ (**b**), and 20 and $50 \,\mathrm {g/L}$ refer to experiments where glucose was added exogenously to the initial condition (**c**)

Modeled overall carbohydrate conversion, calculated by Eq. 45, is compared to the experimental data (calculated in the same way) in Fig. 2. As a derived quantity, conversion amplifies the experimental uncertainties and highlights the differences between predicted and experimental values. We observe that the experimental conversion has plateaued by the end of the experiment (168 h), while the modeled conversions are continuing to increase. While better long-term agreement could be obtained by reducing the adsorption of enzyme to recalcitrant glucan (relative to facile), the agreement would suffer at earlier times. The current model parameters achieve a good fit for the overall conversion profiles, effectively capturing the macroscopic phenomenon of hydrolysis-rate slowdown.

It should be noted that these model coefficients should not be considered to be universal coefficients nor to correspond precisely to detailed low-level physical mechanisms (such as the processive rate of cellulose digestion by CBH I). Because enzyme activity is lumped, these should be understood as phenomenological estimates of this particular system (substrate and enzyme). Additionally, the model parameters resulting from the best-fit approach are closely tied to model assumptions. For example, in this work, we assume that the kinetic rate coefficients for deconstruction are identical for facile and recalcitrant glucan, and that the difference in observed rate is due to reduced accessibility of the recalcitrant cellulose. Flipping these assumptions, such that rate coefficients are allowed to be independent while accessibility is assumed to be identical, will produce a similar fit to experimental data, but with significantly different values for the parameters.

Despite these qualifications, the model variables and parameters are associated with distinct physico-chemical phenomena and can guide researchers performing integrated pretreatment and EH development. Various pretreatment technologies improve the enzymatic digestibility of biomass, but the mechanisms by which the material is made more digestible can, and does, vary. For example, lignocellulose may be made more digestible by increasing delamination of cell walls or by extraction of hemicellulose [5, 15]. Our model captures these two phenomena separately: by increased fraction of facile cellulose (and possibly lower values for $K_{\text{dR}}$) in the case of delamination; and by lower xylan content (which competes for enzyme adsorption) in the case of hemicellulose extraction. For a given system (feedstock, pretreatment, and enzyme cocktail), individual model parameters and initial conditions that are associated with feedstock composition, pretreatment severity, or enzyme dosing may be varied, and the enzymatic-hydrolysis outcome can be predicted with reasonable quantitative confidence. Further, we can use the reaction model to predict outcomes in different reactor systems, as shown in the next section.

### Application to continuous EH data

**Fig. 3:**
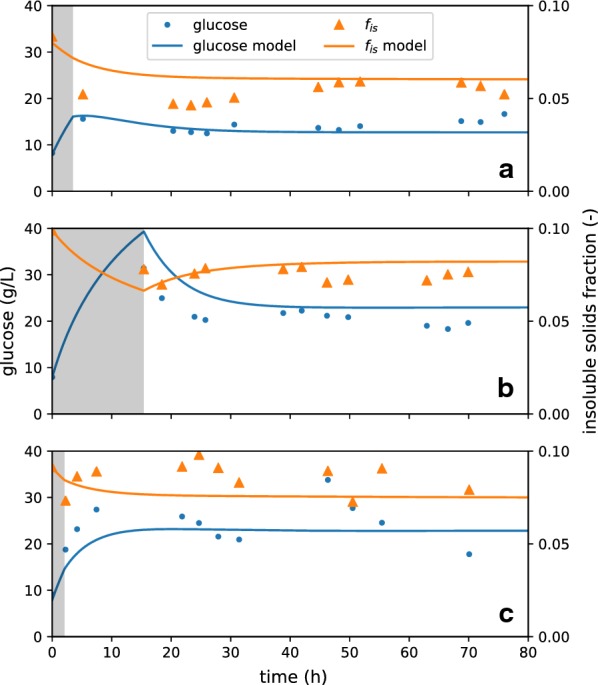
Comparison of continuous EH data to model prediction. Three continuous enzymatic-hydrolysis experiments were performed at $5\%$ (**a**), $7.5\%$ (**b**), and $8.5 \%$ (**c**) target insoluble solids concentration. A period of batch hydrolysis (gray) was performed before the various reactor streams (solids and enzyme feed, membrane filtration, and purge stream) were initiated to decrease the time for the reactor to reach steady state. The data are compared to a model prediction based on parameters generated by the batch experiments and the measured flow rates of the reactor system

A series of continuous EH (CEH) experiments were conducted at several targeted insoluble solids concentrations ($5\%$, $7.5\%$, and $8.5\%$) for up to 72 h of run-time. After an initial batch-startup phase, the flow rates in and out of the reactor were controlled and measured using an Opto 22 (Temecula, CA) automation system. Samples were collected at regular intervals and tested for sugar concentration in the liquor as well as insoluble solids concentration. Mass-balance calculations based on these flow rates were coupled with the kinetics model, using parameters from the batch fit, and the concentration of sugars, solids, and constituents inside the reactor was predicted (Fig. 3). Target solids feed rate was set to maintain a constant feed-weighted residence time across runs, and enzyme was added in solution with a buffer to maintain a constant enzyme loading at a pH of 5. The enzyme solution also acted as a makeup water feed, where water was added in proportion to the liquor lost via permeate, balanced somewhat with deconstruction of solids to liquids, so as to achieve the targetted $f_{\text{is}}$. However, due to handling issues discussed below, the actual feed rates sometimes varied from their setpoints. The measured flow rates and other values relevant to the continuous EH experiments are listed in Table 2.

**Table 2:** Run conditions for each CEH experiment

	Units	I	II	III
Target reactor $f_{\text{is}}$	–	$5\%$	$7.5\%$	$8.5\%$
Enzyme loading	mg/g	10	10	10
Enzyme solution feed rate	kg/h	0.138	0.072	0.054
Enzyme feed concentration	g/L	0.89	2.7	3.6
PT slurry feed rate	kg/h	0.42	0.426	0.348
PT slurry $f_{\text{is}}$	–	$5\%$	$7.5\%$	$10\%$
Permeate rate	kg/h	0.276	0.222	0.152
Purge rate	kg/h	0.282	0.276	0.250
Nominal residence Time	h	17.7	18.1	20.0

Reasonable quantitative agreement with soluble-glucose and insoluble solids data was achieved. For both the $5\%$ and $7.5\%$ targeted $f_{\text{is}}$ experiments, the steady-state model predictions lie close to the experimental data. For the $8.5\%$ data, there is considerably more variatiability in the data, but the model predictions are nonetheless within the observed range of glucose and concentration and $f_{\text{is}}$.

There are some places where the agreement could be improved, especially during the transition from batch to continuous operation, but we think these discrepancies are more likely due to experimental errors rather than due to inadequacies of the model. It should be noted that, as insoluble solids increase, difficulty in handling the material streams likewise increases. Thus, for the higher $f_{\text{is}}$ experiments, the feed, permeate, and purge stream rates are considerably more variable. However, for simplicity, the mass-balance terms imposed for the kinetic model use only static-stream flow rates. These flow rates were determined by the median enzyme solution, solids feed, and permeate flow rates, as these were the most stable measurements available. The system is assumed to maintain a constant mass, and thus the purge rate was calculated from the other flow rates to enforce that assumption.

The ability to quantitatively predict $f_{\text{is}}$ is important. While sugar conversion is of course the desired goal of the enzymatic-hydrolysis process, the rheology of these process slurries are nonlinear functions of the insoluble solids concentrations, where an increase in $f_{\text{is}}$ by 5% can result in an increase in the yield stress by an order of magnitude [30]. The rheology in turn impacts the costs of slurry handling (mixing, pumping, etc.), and in some cases may necessitate different reactor technologies.

Overall, reasonable quantitative agreement was observed between the model predictions (generated by only batch experimental information) and continuous EH data. This agreement between orthogonal data sets supports the simplified phenomenological modeling approach we propose—modeling this reactor would be computationally inaccessible with a more detailed, mechanistic model. It should be noted that residence times (calculated as the volume of the reactor divided by the purge rate) of the CEH experiments were relatively short (18 to 28 h), resulting in overall low conversion. Thus, in the model simulations, these CEH conditions did not result in significant digestion of recalcitrant glucan, and hence the apparent hydrolysis-rate slowdown observed in the batch system was less significant for the CEH experiments. The operation of CEH-CSTRs in sequence, where the purge of the first reactor is the fed to the second and so on, would be a more rigorous challenge to the model. Nonetheless, the batch and CEH experiments presented here sufficiently validate the multi-component substrate (glucan, xylan, lignin) parts of the model, while our previous work, in which the kinetics model was coupled to CFD [27], further supports the two-phase glucan part of the model.

### Sensitivity analysis

**Fig. 4:**
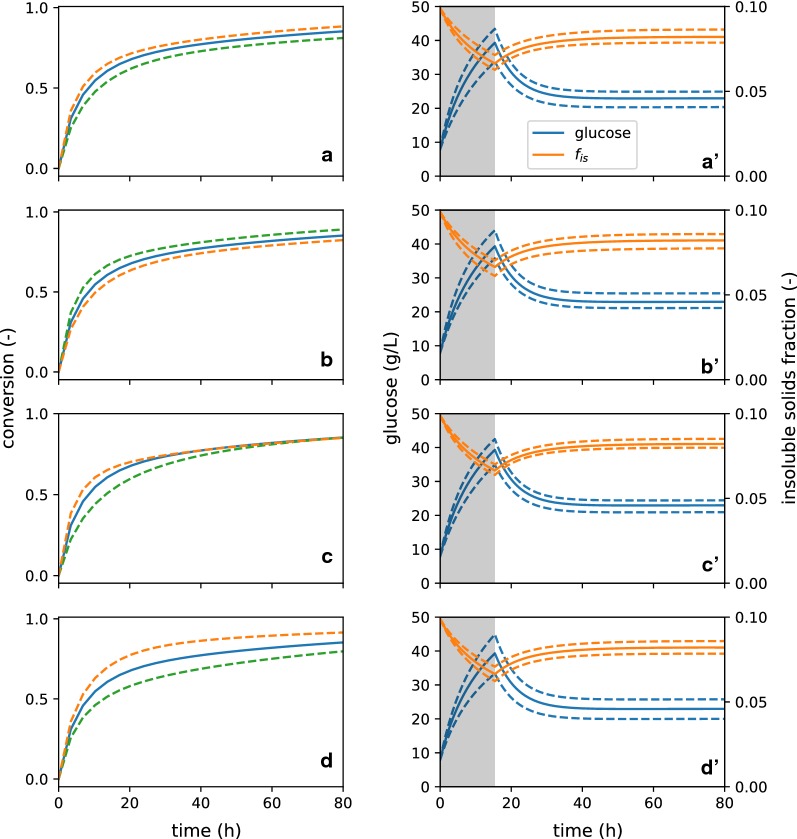
Sensitivity of batch glucose predictions to selected model parameters. Several parameters are varied by $30\%$ upwards (dashed, green) and downwards (dashed, orange) to probe the sensitivity of glucose production to model parameters. The conditions of the experimental reference case were selected as the conditions of the model, and the model here predicts batch data. These parameters are **a** $k_\text{R}$, **b** $\kappa _{\text{Rs}}$, **c** $\kappa _{\text{RF}}$ , and **d** $y_{\text{F},0}$. Additionally, the sensitivity of continuous EH predictions to the same parameters variations is shown in **a′**–**d′**

A few key parameters were systematically varied to demonstrate model features, and the predicted total biomass-conversion (sum of glucan and xylan conversion) was calculated (Fig. 4). The reference-case scenario is defined according to the fit performed above, under the same conditions ($f_{\text{is},0}$, etc.) associated with the experimental reference case, and variations were performed by increasing and decreasing parameter values by $30\%$.

First, the rate of glucose production by adsorbed enzyme ($k_\text{F}$ and $k_\text{R}$) and the degree of competitive adsorption by soluble sugars ($\kappa _{\text{Rs}}$) are considered (Fig. 4a, b). Both parameters affect hydrolysis rate directly (recall that a substantial amount of xylose is in the initial liquor), and that impact continues through the rest of the batch hydrolysis. It is interesting to note that the sensitivity of $k_\text{R}$ and $\kappa _{\text{Rs}}$ are similar—the observed overall rate of conversion is as sensitive to the inhibition to sugars as it is to the kinetics-rate coefficient, at least for the materials and reaction conditions tested. Note also that decreasing the value of $\kappa _{\text{Rs}}$ increases glucose production, following our formalism that $\kappa _{\text{ij}}$ provides a measure of the relative strength of enzyme adsorption between two substrates of interest; for $\kappa _{\text{Rs}}$, it is the relative adsorption between recalcitrant cellulose and competitively inhibiting soluble sugars, where higher values indicate more adsorption to the soluble sugars. This formalism is helpful in analyzing the impact of the respective adsorption parameters.

Next, we consider the impact of $\kappa _{\text{RF}}$, which measures the degree of inaccessibility of the recalcitrant cellulose (Fig. 4c). Increasing $\kappa _{\text{RF}}$ increases relative adsorption onto facile cellulose, which increases initial rates, and vice verse for decreasing $\kappa _{\text{RF}}$. Due to the formulation of the model, this parameter does not substantially impact final rate. Adsorption extents of all species (facile glucan, xylan, soluble sugars, etc.) are related to adsorption on recalcitrant cellulose (via $K_{\text{dR}}$), which sets the base adsorption level. Therefore, in order to modify the final rate in relation to the initial rate, $\kappa _{\text{RF}}$ and $k_{\text{R}}$ must be modified in concert. Finally, it is important to note that, in this fit scenario ($K_{\text{dR}} = 0.05$), nearly all of the enzyme is adsorbed to substrates and inhibitors; therefore, sugar production rate is insensitive to small changes (less than an order of magnitude) in $K_{\text{dR}}$.

Finally, we consider the influence of the partitioning of cellulose between facile and recalcitrant populations ($y_{\text{F}0}$, Fig. 4d). We observe that the initial rate of carbohydrate digestion is the same for all runs. However, with an increased facile population, the extent of conversion before slowdown is increased, and vice versa. This confirms that the division between facile and recalcitrant cellulose has a significant impact on conversion outcomes.

We may also examine the impacts of changing these same model parameters on the predictions for sugar and insoluble solids profiles during CEH (Fig. 4a′–d′). For each of the parameters, the glucose produced in the startup phase are affected, and that impact carries monotonically into the asymptotic glucose concentration in the reactor. Modifying these parameters has a similar influence on $f_{\text{is}}$, which tracks closely with conversion extent. These results again emphasize the portability of this model between reaction contexts. After determining a set of model coefficients for a particular feedstock-pretreatment-enzyme system, engineers may use the model to evaluate how to adjust process conditions (e.g., CEH feed or permeate rate) in response to limited changes to feedstock properties and pretreatment conditions.

## Conclusions

A phenomenological model was developed in order to predict outcomes of enzymatic hydrolysis of lignocellulose for various reactor configurations and operating conditions. A series of batch experiments were performed with varying initial conditions (solids, enzyme, and sugar concentrations), and best-fit model parameters were determined using nonlinear constrained least-squares. The model achieved a good fit for overall sugar yield and insoluble solids concentration, as well as for the reduced rate of sugar production over time. A second set of experiments were performed using membrane reactors in which the hydrolysis reactions were performed in a continuous mode. Quantitative agreement was observed between these continuous enzymatic-hydrolysis experiments and model predictions. Finally, the sensitivity of the model to its parameters was explored and discussed.

We emphasize again that the phenomena represented by the model correspond to behaviors that emerge from clusters of mechanisms, and hence a set of model coefficients are unique to the substrate and the enzyme system. Nonetheless, we think that this modeling approach finds the right balance between model complexity and computational efficiency. After fitting our proposed phenomenological model to carefully performed laboratory batch experiments, the model may be applied to novel reactor schema and implemented in CFD simulations. Computationally intensive mechanistic models have their use for exploring detailed physico-chemical phenomena of enzymatic hydrolysis of lignocellulose—for example, evaluating the trade-offs between varying the amounts of specific component enzymes. As mechanistic models and their numerical solution become more sophisticated, they may also become suitable for engineering purposes. In the meantime, phenomenological models, like the one proposed here, have broad application to reactor design, scale-up, and process optimization.

## Materials and methods

### Pretreated corn stover

Corn stover was obtained from Idaho National Laboratory (Idaho Falls, ID, USA) where it was knife-milled using a $13 \,\mathrm {mm}$ rejection screen. This feedstock was then deacetylated by soaking in $0.4 \% \,\mathrm {w/w}$ sodium hydroxyide at $80\,^\circ \mathrm {C}$ for 2 h. It was then rinsed with water and soaked in $1.0 \% \,\mathrm {w/w}$ sulfuric acid. Free water was removed using a screw press, taking the slurry to $50\%$ insoluble solids. Finally, the acid-impregnated slurry was thermochemically hydrolyzed at $160\,^\circ \mathrm {C}$ for 15 min in a $500 \,\mathrm {kg/day}$ horizonal reactor (Metso, Inc, Norcross, GA) [24].

The material was then neutralized to pH 5.0 using $90 \%$ sodium hydroxide, and the final insoluble solids fraction was measured and found to be $f_{\text{is}} = 0.23$. Dilutions of this material with water and sodium citrate buffer were used as the basis for all experiments, and the solids are composed of $62\%$ glucan, $6\%$ xylan, $22\%$ lignin, and $10\%$ unknown structural carbohydrates and ash [26].

### Batch enzymatic hydrolysis

A series of batch enzymatic-hydrolysis experiments were performed to estimate rate and other parameter values for the model. A reference condition was performed in duplicate with initial insoluble solids of $10\%$ and an enzyme loading of $20 \,\mathrm {mg/g}$ Cellulose (CTEC3, Novozymes). CTEC3 is a commercial cellulase preparation, containing a proprietary mixture of enzymes including cellulases, hemicellulases, and glucosidases. Additional experiments were performed at the similar conditions but with varied initial insoluble solids loading, enzyme loading, and initial glucose concentration (glucose was added directly to the initial slurry). A complete list of experimental conditions is provided in Table 3.

**Table 3:** Batch experimental conditions

$f_{\text{is}}$	$\lambda$ (mg-CTEC3/g-cellulose)	Added glucose (g/L)
0.10	20	0
0.075	20	0
0.05	20	0
0.10	15	0
0.10	10	0
0.10	20	20
0.10	20	50

The condition of the first row is considered the reference condition (“ref”) and was performed in duplicate

These experiments were performed in $50 \,\mathrm {mL}$ roller bottles [23]. Samples were taken for soluble sugar analysis by HPLC [26] at 4, 8, 24, 28, 72, 120, and 168 h, and samples for insoluble solids measurement [35] were taken at 24 and 168 h. Soluble dimer (cellobiose) and oligimer concentrations were negligible and therefore are not reported. All experiments were performed at $50\,^\circ \mathrm {C}$ with a pH of 5.0 buffered with $100 \,\mathrm {mM}$ sodium citrate.

### Continuous enzymatic hydrolysis

**Fig. 5:**
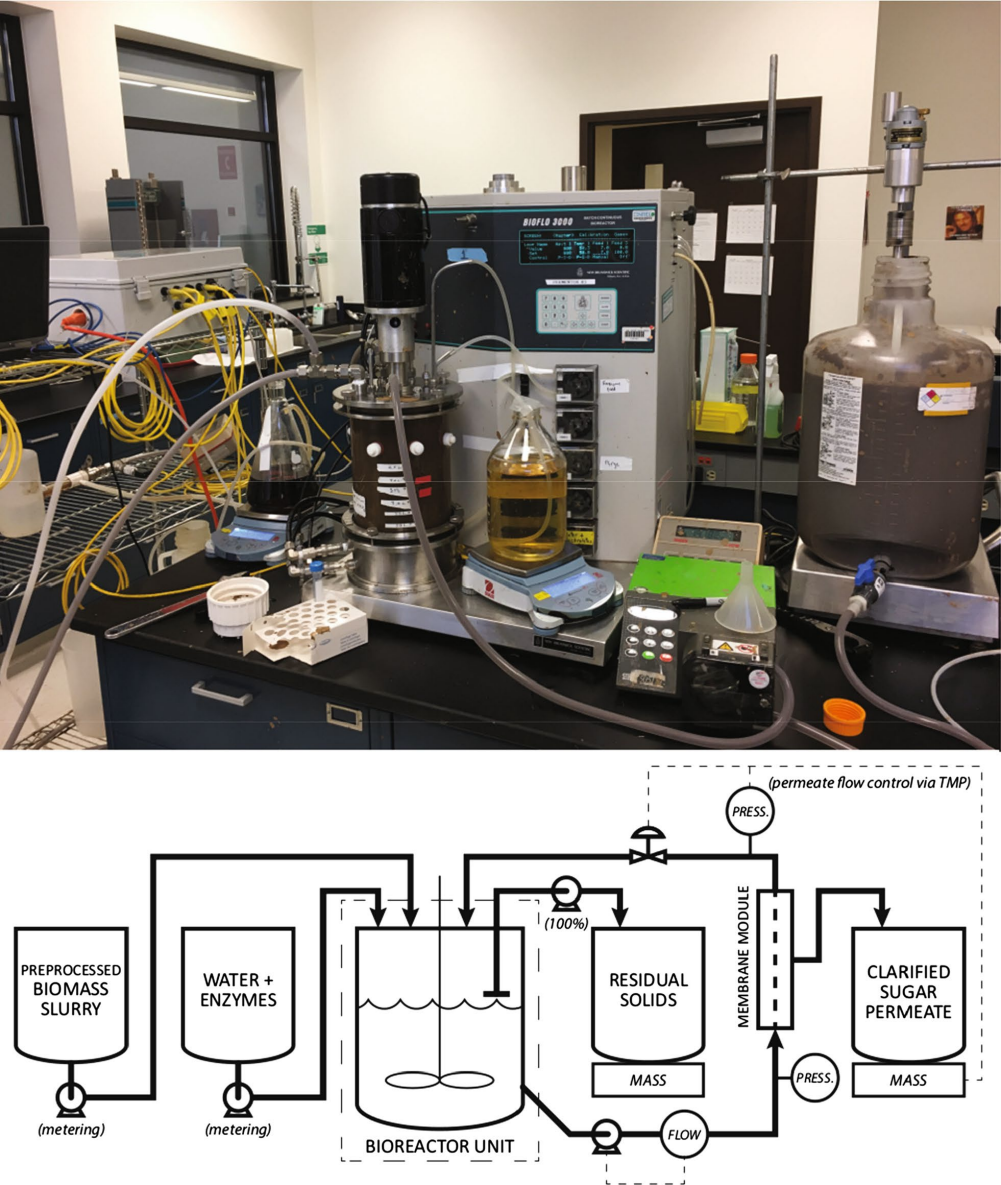
Continuous EH experimental apparatus. The experimental apparatus (top) and process-flow diagram (bottom) for the continuous EH experiments

A bench-top-scale apparatus was used to test continuous enzymatic hydrolysis (CEH) at different targeted insoluble solids loadings ($5 \%$, $7.5 \%$, and $8.5 \%$) (Fig. 5) [31]. A $5 \,\mathrm {L}$ vertically stirred tank (BioFlo 3000, New Brunswick Scientific, Inc.) with a marine impeller (2 in diameter, 300 RPM) was used as the reaction vessel. Temperature in this vessel (as measured by a probe inside the vessel) was maintained at $50\,^\circ \mathrm {C}$ by a hot-water jacket. Liquor was removed from the system by cross-flow filtration (Koch M180, MWCO of $100 \,\mathrm {kDa}$, 0.5 in OD, $0.0122 \,\mathrm {m^2}$ surface area); experiments showed that this membrane retains approximately $50\%$ of soluble enzymes. Fresh substrate, fresh enzyme (CTEC3, Novozymes), and buffer (pH 5.0, $100 \,\mathrm {mM}$) were added to the reactor via peristaltic pumps, and a purge line maintained the vessel at a constant fill volume. Run-time variables were recorded and controlled using a data acquisition and control system.

After an initial startup period, where pretreated solids were allowed to hydrolyze in a batch-like mode, the CEH system was allowed to run for approximately 3 days, with slurry samples taken from the reactor vessel three to four times a day, and the insoluble solids and soluble sugars of the slurry were measured.

### Enzymatic-hydrolysis model

The molar concentrations of the two cellulose (glucan) substrates are denoted as $\tilde{c}_{\text{GF}}$ and $\tilde{c}_{\text{GR}}$, for the facile (F) and recalcitrant (R) populations, respectively. These terms represent the molar concentrations of glucan monomers, not the cellulose polymers. The glucose product concentration is denoted $c_\text{g}$. Because of the insoluble nature of the cellulose substrate, a whole-slurry volume basis is used for glucan concentrations, i.e., a tilde over a term (${\tilde{c}}_{\text{GR}}$) denotes a total-slurry-volume basis for concentration ($\mathrm {\frac{kmol}{m^3\ slurry}}$), whereas the absence of a tilde ($c_\text{g}$) denotes a liquid-volume basis ($\mathrm {\frac{kmol}{m^3\ liquid}}$), to be used for soluble species. Likewise, there are molar concentrations for xylan (X), lignin (L), xylose (x), and soluble lignin (sL). The total concentration of soluble sugars (ss) is the sum of glucose and xylose concentrations.

Slurry- and liquid-basis molar concentrations may be related by

1 $$\begin{aligned} {\tilde{c}}_i = \epsilon _\text{l} c_i, \end{aligned}$$

where $\epsilon _\text{l}$ is the *volume* fraction of liquid in the slurry. Similarly, the mass fraction of species *i* relative to the slurry ($f_i$) and the molar concentration may be related by

2 $$\begin{aligned} f_i = \frac{M_{\text{W},i} {\tilde{c}}_i}{\rho _\text{T}} = \frac{M_{\text{W},i} \epsilon _\text{l} c_i}{\rho _\text{T}} \end{aligned}$$

where $M_{\text{W},i}$ is the molecular weight of species *i*.

The liquid volume fraction is related to the mass fraction of liquid ($f_\text{l}$), liquid density ($\rho _\text{l}$), and total slurry density ($\rho _\text{T}$) by

3 $$\begin{aligned} \epsilon _\text{l} = \frac{\rho _\text{T}}{\rho _\text{l}}f_\text{l}, \end{aligned}$$

where $f_\text{l} = 1 - f_{\text{is}}$, and $f_{\text{is}} = f_{\text{GF}} + f_{\text{GR}} + f_\text{X} + f_\text{L}$ is the mass fraction of insoluble solids. The total slurry density is related to the skeletal density of insoluble solids ($\rho _{\text{is}}$) by

4 $$\begin{aligned} \rho _\text{T} = \left( \frac{f_{\text{is}}}{\rho _{\text{is}}} + \frac{f_\text{l}}{\rho _\text{l}}\right) ^{-1}. \end{aligned}$$

The slurry density may be approximated by that of the liquid for low solids concentrations and for densities of solids close to that of the liquid. Specifically, $\rho _\text{T} \approx \rho _\text{l} = 1000\,\mathrm {kg/m^3}$ (density of water, here), resulting in less than $5\%$ error when $\rho _{\text{is}} < 1400 \,\mathrm {kg/m^3}$ and $f_{\text{is}} < 0.15$.

#### Enzyme adsorption and inhibition

**Fig. 6:**
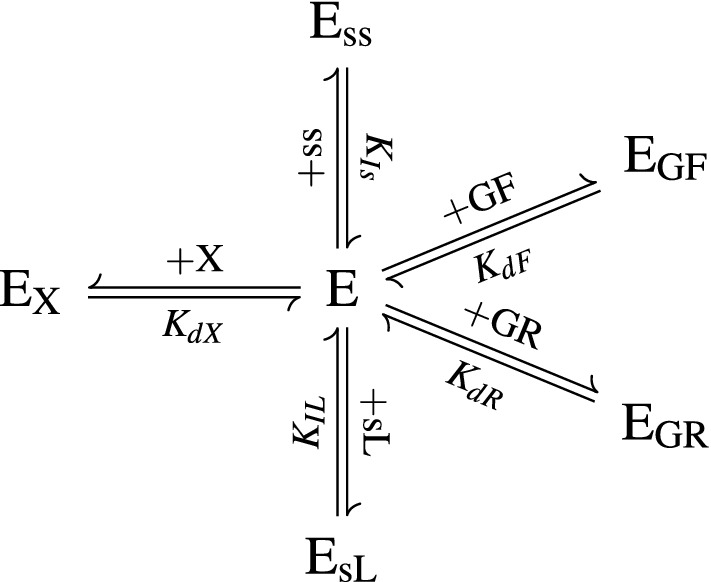
Adsorption and inhibition of the enzymes in the kinetics model

The enzymes in the cocktail are treated collectively, and the total molar concentration is denoted $\tilde{c}_{\text{ET}}$. The enzymes partition between being free in solution ($c_{\text{Ef}}$), adsorbed to each glucan substrate ($\tilde{c}_{\text{EGF}}$ and $\tilde{c}_{\text{EGR}}$), adsorbed to xylan ($\tilde{c}_{\text{EX}}$), inhibited by soluble lignin ($c_{\text{EsL}}$), and inhibited by soluble sugars ($c_{\text{Ess}}$) (Fig. 6), so that

5 $$\begin{aligned} \tilde{c}_{\text{ET}} = \tilde{c}_{\text{Ef}} + \tilde{c}_{\text{EGF}} + \tilde{c}_{\text{EGR}} + \tilde{c}_{\text{EX}} +\tilde{c}_{\text{EsL}} + \tilde{c}_{\text{Ess}}. \end{aligned}$$

The equilibrium relationships for adsorption and inhibition are given by

6 $$\begin{aligned} K_{\text{dF}} = \frac{c_{\text{Ef}} \tilde{c}_{\text{GF}}}{\tilde{c}_{\text{EGF}}}, \end{aligned}$$

7 $$\begin{aligned} K_{\text{dR}} = \frac{c_{\text{Ef}} \tilde{c}_{\text{GR}}}{\tilde{c}_{\text{EGR}}}, \end{aligned}$$

8 $$\begin{aligned} K_{\text{dX}} = \frac{c_{\text{Ef}} \tilde{c}_{\text{X}}}{\tilde{c}_{\text{EX}}}, \end{aligned}$$

9 $$\begin{aligned} K_{\text{IL}} = \frac{c_{\text{Ef}} c_{\text{sL}}}{c_{\text{EsL}}}, \end{aligned}$$

10 $$\begin{aligned} K_{\text{Is}} = \frac{c_{\text{Ef}} c_{\text{ss}}}{c_{\text{Ess}}}, \end{aligned}$$

The equilibrium terms, $K_{i}$, are *dissociation* coefficients—this means that lower values of $K_{i}$ result in higher adsorption/inhibition.

Combining Eqs. 5–10 results in relationships for the concentration of enzymes adsorbed to xylan and facile and recalcitrant cellulose. Including a few convenience terms:

11 $$\begin{aligned} \kappa _{\text{RF}}&= \frac{K_{\text{dR}}}{K_{\text{dF}}}, \end{aligned}$$

12 $$\begin{aligned} \kappa _{\text{RX}}&= \frac{K_{\text{dR}}}{K_{\text{dX}}}, \end{aligned}$$

13 $$\begin{aligned} \kappa _{\text{RL}}&= \frac{K_{\text{dR}}}{K_{\text{IL}}}, \end{aligned}$$

14 $$\begin{aligned} \kappa _{\text{Rs}}&= \frac{K_{\text{dR}}}{K_{\text{Is}}}, \end{aligned}$$

15 $$\begin{aligned} \lambda _{\text{FR}}&= \frac{\tilde{c}_{\text{GF}}}{\tilde{c}_{\text{GR}}}, \end{aligned}$$

16 $$\begin{aligned} \lambda _{\text{XR}}&= \frac{\tilde{c}_{\text{X}}}{\tilde{c}_{\text{GR}}}, \end{aligned}$$

we have

17 $$\begin{aligned} \tilde{c}_{\text{EGR}}&= \frac{\tilde{c}_{\text{ET}}}{{\mathcal {D}}}, \end{aligned}$$

18 $$\begin{aligned} \tilde{c}_{\text{EGF}}&= \frac{\kappa _{\text{RF}}\lambda _{\text{FR}}\tilde{c}_{\text{ET}}}{{\mathcal {D}}}, \end{aligned}$$

19 $$\begin{aligned} \tilde{c}_{\text{EX}}&= \frac{\kappa _{\text{RX}}\lambda _{\text{XR}}\tilde{c}_{\text{ET}}}{{\mathcal {D}}}, \end{aligned}$$

where

20 $$\begin{aligned} {\mathcal {D}} = 1 + \kappa _{\text{RF}}\lambda _{\text{FR}} + \kappa _{\text{RX}}\lambda _{\text{XR}} + \frac{\epsilon _\text{l}}{\tilde{c}_{\text{GR}}} \left( K_{\text{dR}} + \kappa _{\text{RL}}c_{\text{sL}} + \kappa _{\text{Rs}}c_{\text{ss}}\right) . \end{aligned}$$

The $\kappa _i$ terms not only help to provide compact equations for adsorption, but they are also useful to use directly as model coefficients. The adsorption parameter for enzymes on recalcitrant glucan, $K_{\text{dR}}$, can be thought to set the scale for enzyme adsorption, while the $\kappa _i$ relate the adsorption on the other species to the adsorption on recalcitrant glucan.

Rather than using hydrolysis-rate coefficients (introduced in the next section) to effect biphasic behavior, we instead construct the model to emphasize that the structure of recalcitrant cellulose is the mechanism for the emergent biphasic behavior. This is accomplished by having different desorption coefficients for recalcitrant and facile glucan, where $K_{\text{dF}} < K_{\text{dR}}$ ($\kappa _{\text{RF}} > 1$) results in more enzymes being adsorbed to facile glucan than recalcitrant. To further illustrate how this approach is related to structure, consider that not all recalcitrant glucose is accessible to enzy‘me. We presume that a simple proportionality constant ($\alpha$) can be used to relate the accessible portion of recalcitrant glucan to the total concentration of recalcitrant glucan:

21 $$\begin{aligned} \tilde{c}_{\text{GRA}}= \alpha \cdot \tilde{c}_{\text{GR}},\text { where } \alpha \in [0,1]. \end{aligned}$$

Thus, an equilibrium coefficient for enzyme adsorption to accessible recalcitrant glucan is given by

22 $$\begin{aligned} K_{\text{dRA}} = \frac{c_{\text{Ef}} \tilde{c}_{\text{GRA}}}{\tilde{c}_{\text{EGR}}} = \frac{c_{\text{Ef}} \alpha \tilde{c}_{GR}}{\tilde{c}_{\text{EGR}}} = \alpha K_{\text{dR}}. \end{aligned}$$

Because these equilibrium terms correspond to the relative rates of adsorption and desorption of the enzyme on the substrate surface, we may assume that $K_{\text{dF}} = K_{\text{dRA}}$. Thus,

23 $$\begin{aligned} \kappa _{\text{RF}} = \frac{K_{\text{dR}}}{K_{\text{dF}}} = \frac{K_{\text{dRA}}}{\alpha K_{\text{dF}}} = \alpha ^{-1}, \end{aligned}$$

which is to say that $\kappa _{\text{RF}}$ can be thought of as a term capturing the substrate accessibility of the recalcitrant cellulose, at least for the linearly proportional accessibility model assumed here.

#### Reaction kinetics

The molar reaction rates ($\mathrm {\frac{kmol}{m^3\,h}}$), on a total slurry basis, of digestion for the substrates are given by

24 $$\begin{aligned} - {\tilde{r}}_{\text{GR}}&= k_\text{R}\tilde{c}_{\text{EGR}}, \end{aligned}$$

25 $$\begin{aligned} - {\tilde{r}}_{\text{GF}}&= k_\text{F}\tilde{c}_{\text{EGF}}, \end{aligned}$$

26 $$\begin{aligned} - {\tilde{r}}_\text{X}&= k_\text{X}\tilde{c}_{\text{EX}}, \end{aligned}$$

27 $$\begin{aligned} {\tilde{r}}_{\text{g}}&= -({\tilde{r}}_{\text{GR}} + {\tilde{r}}_{\text{GF}}), \end{aligned}$$

28 $$\begin{aligned} {\tilde{r}}_{\text{x}}&= -{\tilde{r}}_{\text{X}}, \end{aligned}$$

where $k_i$ are the reaction-rate coefficients.

We have found that carbohydrate digestion alone does not fully account for the reduction in $f_{\text{is}}$, as previously observed by Roche et al. [23]. This suggests that lignin is also solubilized by the enzymatic reaction, although, to our knowledge, the mechanisms for lignin solubilization are not understood and likely quite complex. We suggest that lignin is structurally integrated with carbohydrates such that it is liberated as those carbohydrates are solubilized. Low-molecular-weight lignin-polymers may become solubilized as they are liberated, such that $f_{\text{is}}$ is reduced by lignin solubilization as well as carbohydrate conversion. This model captures this phenomenon with a simple rate equation:

29 $$\begin{aligned} {\tilde{r}}_\text{L}&= k_\text{L}\tilde{c}_\text{L}\left( {\tilde{r}}_{\text{GR}} + {\tilde{r}}_{\text{X}} \right) , \end{aligned}$$

30 $$\begin{aligned} {\tilde{r}}_{\text{sL}}&= -{\tilde{r}}_\text{L}. \end{aligned}$$

Initial conditions are commonly given by the initial fraction of insoluble solids $f_{\text{is}0}$, the initial fraction of each insoluble species as part of the insoluble solids, $w_i$, the initial concentration of soluble species, $\rho _i$, and the enzyme loading $\lambda _\text{E} = f_{\text{ET}}/f_{\text{G}0}$ (where $f_{\text{ET}}$ is the fraction of total enzyme). These parameters are all easily measured or directly controlled. It is also necessary to specify the initial facile fraction $y_{\text{F}0} = f_{\text{GF}0}/f_{\text{G}0}$. Conceptually, this term may be related to cellulose crystallinity, though this connection is loose [18], or to the structure of the remaining cell wall. In this context, it may be treated as a model parameter associated with biomass that may be fit to experimental data, along with the adsorption and rate parameters.

### Model summary and application to reactors

In summary, enzymes in solution are partitioned between solid-phase substrate (glucan and xylan) and liquid-phase inhibitors. The concentrations of adsorbed and inhibited enzymes are determined through algebraic equilibrium relationships (Eqs. 17–19). Adsorbed enzyme-substrate complexes then convert solid-phase substrate into soluble sugars by first-order kinetics (Eqs. 24–28), and lignin solubilization is modeled as dependent on the rate of recalcitrant glucan and xylan conversion (Eqs. 29 and 30).

It is generally convenient to use normalized mass-based reaction-rate terms (${\mathcal {R}}_i$, with units $\mathrm {\frac{kg}{kg\,h}}$), as these are quickly relatable to measureable quantities. For the batch reactor, the mass-based reaction rates are given by

31 $$\begin{aligned} {\mathcal {R}}_{\text{GR}}&= \frac{M_{\text{W,G}}}{\rho _\text{T}}{\tilde{r}}_{\text{GR}}, \end{aligned}$$

32 $$\begin{aligned} {\mathcal {R}}_{\text{GF}}&= \frac{M_{\text{W,G}}}{\rho _\text{T}}{\tilde{r}}_{\text{GF}}, \end{aligned}$$

33 $$\begin{aligned} {\mathcal {R}}_\text{X}&= \frac{M_{\text{W,X}}}{\rho _\text{T}}{\tilde{r}}_\text{X}, \end{aligned}$$

34 $$\begin{aligned} {\mathcal {R}}_\text{L}&= \frac{M_{\text{W,L}}}{\rho _\text{T}}{\tilde{r}}_\text{L}, \end{aligned}$$

35 $$\begin{aligned} {\mathcal {R}}_\text{g}&= \frac{M_{\text{W,g}}}{\rho _\text{T}}{\tilde{r}}_\text{g}, \end{aligned}$$

36 $$\begin{aligned} {\mathcal {R}}_\text{x}&= \frac{M_{\text{W,x}}}{\rho _\text{T}}{\tilde{r}}_\text{x}, \end{aligned}$$

37 $$\begin{aligned} {\mathcal {R}}_\text{l}&= \frac{M_{\text{W,x}}}{\rho _\text{T}}{\tilde{r}}_\text{l}, \end{aligned}$$

where $M_{\text{W},i}$ is the molecular weight of species *i* (with the monomer molecular weight used for our polymers). Lignin is a special case, as it is a polymer with diverse monomers (unlike glucan and xylan), and there is therefore a range of monomer molecular weights associated with lignin. However, it is outside the scope of this model to disentangle lignin chemistry, so we choose $200 \,\mathrm {Da}$ as a representative molecular weight, which is within the range (188 to $211 \,\mathrm {Da}$) reported by Chua and Wayman [4].

### Batch reaction

For a well-mixed batch reaction with no time-variable inlets and outlets, the rates of change of the species concentrations are proportional to the mass-based reaction rates:

38 $$\begin{aligned} \frac{\mathrm {d}f_{\text{GR}}}{\mathrm {d}t}&= {\mathcal {R}}_{\text{GR}}, \end{aligned}$$

39 $$\begin{aligned} \frac{\mathrm {d}f_{\text{GF}}}{\mathrm {d}t}&= {\mathcal {R}}_{\text{GF}}, \end{aligned}$$

40 $$\begin{aligned} \frac{\mathrm {d}f_\text{X}}{\mathrm {d}t}&= {\mathcal {R}}_\text{X}, \end{aligned}$$

41 $$\begin{aligned} \frac{\mathrm {d}f_\text{L}}{\mathrm {d}t}&= {\mathcal {R}}_\text{L}, \end{aligned}$$

42 $$\begin{aligned} \frac{\mathrm {d}f_\text{g}}{\mathrm {d}t}&= {\mathcal {R}}_\text{g}, \end{aligned}$$

43 $$\begin{aligned} \frac{\mathrm {d}f_\text{x}}{\mathrm {d}t}&= {\mathcal {R}}_\text{x}, \end{aligned}$$

44 $$\begin{aligned} \frac{\mathrm {d}f_{\text{sL}}}{\mathrm {d}t}&= {\mathcal {R}}_{\text{sL}}. \end{aligned}$$

The total enzymatic conversion of carbohydrates in a batch reaction is calculated by

45 $$\begin{aligned} X_\text{t} = \frac{ r_\text{g} (f_\text{g} - f_{\text{g},0}) + r_\text{x} (f_\text{x} - f_{\text{x},0})}{f_{\text{is},0}(w_{\text{g},0} + w_{\text{x},0})}, \end{aligned}$$

where $r_i$ is the ratio of the molecular weight of the polysaccharide monomer to the molecular weight of the hydrolyzed soluble sugar [31].

### Continuous reaction

The CEH system requires coupling the reaction rates with a mass-balance that accounts for the different streams entering and exiting the reactor. As illustrated in Fig. 5, these streams include the solids feed (${\dot{m}}_{s,\mathrm {in}}$), the enzyme addition (${\dot{m}}_{e,\mathrm {in}}$), the membrane-permeate stream (${\dot{m}}_{m,\mathrm {out}}$), and the purge stream (${\dot{m}}_{p,\mathrm {out}}$). The reactor is considered to be constant mass, and so the sum of inputs is equal to the sum of outputs (${\dot{m}}_{s,\mathrm {in}} + {\dot{m}}_{e,\mathrm {in}} = {\dot{m}}_{m,\mathrm {out}} + {\dot{m}}_{p,\mathrm {out}}$). Solid components are introduced to the reactor in the solids feed, exit the reactor in proportion to the solids fraction in the purge feed, and are consumed by reaction:

46 $$\begin{aligned} \frac{\mathrm {d}f_{\text{GR}}}{\mathrm {d}t}&= \frac{ {\dot{m}}_{s,\mathrm {in}} }{m_\text{T}} f_{\text{GR},0} - \frac{ {\dot{m}}_{p,\mathrm {out}} }{m_\text{T}} f_{\text{GR}} + {\mathcal {R}}_{\text{GR}} , \end{aligned}$$

47 $$\begin{aligned} \frac{\mathrm {d}f_{\text{F}}}{\mathrm {d}t}&= \frac{ {\dot{m}}_{s,\mathrm {in}} }{m_\text{T}} f_{\text{GF},0} - \frac{ {\dot{m}}_{p,\mathrm {out}} }{m_\text{T}} f_{\text{GF}} + {\mathcal {R}}_{\text{GF}} , \end{aligned}$$

48 $$\begin{aligned} \frac{\mathrm {d}f_{\text{X}}}{\mathrm {d}t}&= \frac{ {\dot{m}}_{s,\mathrm {in}} }{m_\text{T}} f_{\text{X},0} - \frac{ {\dot{m}}_{p,\mathrm {out}} }{m_\text{T}} f_{\text{X}} + {\mathcal {R}}_{\text{X}} , \end{aligned}$$

49 $$\begin{aligned} \frac{\mathrm {d}f_{\text{L}}}{\mathrm {d}t}&= \frac{ {\dot{m}}_{s,\mathrm {in}} }{m_\text{T}} f_{\text{L},0} - \frac{ {\dot{m}}_{p,\mathrm {out}} }{m_\text{T}} f_{\text{L}} + {\mathcal {R}}_{\text{L}}. \end{aligned}$$

The soluble species are introduced in the feedstock feed, exit in the both the permeate and the purge streams, and are produced by reaction.

50 $$\begin{aligned} \frac{\mathrm {d}f_{\text{g}}}{\mathrm {d}t} =&\frac{ {\dot{m}}_{s,\mathrm {in}} }{m_\text{T}} f_{\text{g},0} - \frac{ {\dot{m}}_{p,\mathrm {out}} }{m_\text{T}} f_\text{g} - \frac{ {\dot{m}}_{m,\mathrm {out}} }{m_\text{T}} \frac{f_\text{g}}{\epsilon _\text{l}} + {\mathcal {R}}_{\text{g}}, \end{aligned}$$

51 $$\begin{aligned} \frac{\mathrm {d}f_{\text{x}}}{\mathrm {d}t} =&\frac{ {\dot{m}}_{s,\mathrm {in}} }{m_\text{T}} f_{\text{x},0} - \frac{ {\dot{m}}_{p,\mathrm {out}} }{m_\text{T}} f_\text{x} - \frac{ {\dot{m}}_{m,\mathrm {out}} }{m_\text{T}} \frac{f_\text{x}}{\epsilon _\text{l}} + {\mathcal {R}}_{\text{x}}, \end{aligned}$$

52 $$\begin{aligned} \frac{\mathrm {d}f_{\text{l}}}{\mathrm {d}t} =&\frac{ {\dot{m}}_{s,\mathrm {in}} }{m_\text{T}} f_{\text{l},0} - \frac{ {\dot{m}}_{p,\mathrm {out}} }{m_\text{T}} f_\text{l}- \frac{ {\dot{m}}_{m,\mathrm {out}} }{m_\text{T}} \frac{f_\text{l}}{\epsilon _\text{l}} + {\mathcal {R}}_{\text{l}}. \end{aligned}$$

Finally, the enzymes are considered. Enzymes enter through the enzyme addition stream, exit through the purge stream, and free enzymes in solution as well as soluble-species-inhibited enzyme are partially rejected by the filter. To test the rejection efficiency of our system (nominal MWCO at $100 \,\mathrm {kDa}$) with respect to our enzyme, we filtered a dilute solution of our enzyme, and then measured total protein in the filtrate and retentate by UV–Vis adsorption at $280 \,\mathrm {nm}$. Our data indicated that the rejection coefficient was approximately $\eta _\text{E} = 0.5$.

53 $$\begin{aligned} \frac{\mathrm {d}f_{\text{ET}}}{\mathrm {d}t} =&\frac{ {\dot{m}}_{e,\mathrm {in}} }{m_\text{T}} f_{\text{E},0} - \frac{ {\dot{m}}_{p,\mathrm {out}} }{m_\text{T}} f_{\text{ET}} \\&- \frac{ {\dot{m}}_{m,\mathrm {out}} }{m_\text{T}} \eta _\text{E} \frac{c_{\text{Ess}}+c_{\text{EsL}}+c_{\text{Ef}}}{\epsilon _\text{l}} \end{aligned}$$

## Data Availability

All data generated or analyzed during this study are included in this published article.

## Variables

$c_i$: concentration of species *i* on a liquid basis ($\mathrm {\frac{kmol}{m^3\ liquid}}$)
$\tilde{c}_i$: concentration of species *i* on total slurry basis ($\mathrm {\frac{kmol}{m^3\ slurry}}$)
$\epsilon_\text{l}$: volume fraction of liquid in the slurry (–)
$f_i$: mass fraction of species *i* in the slurry (–)
$f_{\text{is}}$: mass fraction of insoluble solids in the slurry (–)
$f_{\text{Ris}}$: mass fraction of recalcitrant solids (GR, X, L) in the slurry (–)
$k_\text{R}$, $k_\text{F}$, $k_\text{X}$: molar reaction coefficient of recalcitrant (R) and facile (F) glucan and xylan (X) complexed with enzyme ($\mathrm{h^{-1}}$)
$k_\text{L}$: molar reaction coefficient of lignin, which is defined in relationship to glucan and xylan deconstruction ($\mathrm {\frac{m^2 liquid}{kmol}}$)
$K_{\text{di}}$: dissociation coefficients for reactive solid species (GR, GF, X) ($\mathrm {\frac{kmol}{m^3\ liquid}}$)
$K_{\text{Ii}}$: dissociation coefficients for inhibitory species (soluble sugars and lignin) ($\mathrm {\frac{kmol}{m^3\ liquid}}$)
$\kappa _{\text{Ri}}$: measure of adsorption of enzyme on species *i* relative to adsorption on recalcitrant glucan (–)
$\lambda _{\text{FR}}$, $\lambda _{\text{XR}}$: convenience terms relating concentration of facile glucan, recalcitrant glucan, and xylan (–)
$M_{\text{W},i}$: molecular weight of species *i* (Da). For polymers, this references the molecular weight of the monomer
$\rho _\text{T}$: density of the slurry ($\text{kg/m}^3$)
$\rho _{\text{is}}$: density of the skeletal solids ($\text{kg/m}^3$)
$w_{j}$: mass fraction of solids consisting of species *j* (–)
$X_{j}$: molar conversion of species *j* (–)
$y_{\text{F}0}$: initial mass fraction of the glucan consisting of the facile population (–)

## Species

Ei: Enzyme, complexed with species *i*, or in total ($i=T$)
GR: recalcitrant glucan
GF: facile glucan
X: xylan
L: solid lignin
g: glucose
x: xylose
ss: soluble sugars (glucose and xylose)
sL: soluble lignin
